# Supplementary material for: Non-hysteretic first-order phase transition with large latent heat and giant low-field magnetocaloric effect
Source: Nat Commun. 2018 Jul 26;9:2925. doi: 10.1038/s41467-018-05268-4 (PMC6062548; doi:10.1038/s41467-018-05268-4)
Supplement: Supplementary file 1 — Supplementary Information [file 41467_2018_5268_MOESM1_ESM.pdf]

Supplementary Information for:

Non-hysteretic first-order phase transition with large latent heat and giant low-field magnetocaloric effect

By F. Guillou *et al.*

Supplementary Note 1: Powder x-ray diffraction at room temperature

Powder x-ray diffraction (XRD) experiments were carried out using Mo K_α radiation on Rigaku TTRAX and Cu $K_{\alpha 1}$ radiation on PANalytical X'Pert PRO diffractometers. In both experiments the air sensitivity of the material was addressed in a different manner. For the former, ground particles screened to less than 22 μm in diameter are mixed with petroleum jelly and mounted on a Cu sample holder in an Ar-filled glovebox. The measurements are performed with the sample located in a vacuum chamber with 10^{-5} mbar base pressure. For the second experiments, the particles are spread on a zero-background single crystalline Si sample holder, then hermetically covered by a Kapton film in an Ar glovebox. The measurements are then performed in air using a rotating sample stage. The Rietveld refinement of the powder XRD pattern for Mo K_α is shown in **Supplementary Figure 1**, and the results of both experiments are summarized in **Supplementary Table 1**. The representation of the crystal structure of Eu_2In illustrates the relationship between the different layers of atoms.

	a	b	c	V	x and z			R_p	R_{wp}	χ^2
					Eu-I	Eu-II	In			
Mo K_α	7.4536(6)	5.5822(5)	10.3121(9)	429.06(7)	0.0287(4), 0.7048(3)	0.1781(4), 0.0680(3)	0.2217(5), 0.3929(4)	7.55	9.4	4.55
Cu $K_{\alpha 1}$	7.4527(8)	5.5787(6)	10.313(1)	428.77(9)	0.029(1), 0.706(1)	0.180(1), 0.067(1)	0.225(1), 0.394(1)	5.87	5.33	1.23

Supplementary Table 1: Crystallographic parameters of Eu_2In. Co_2Si-type structure with space group symmetry Pnma, all atoms in 4c positions (*x*, ¼, *z*), derived from two independent powder XRD experiments.

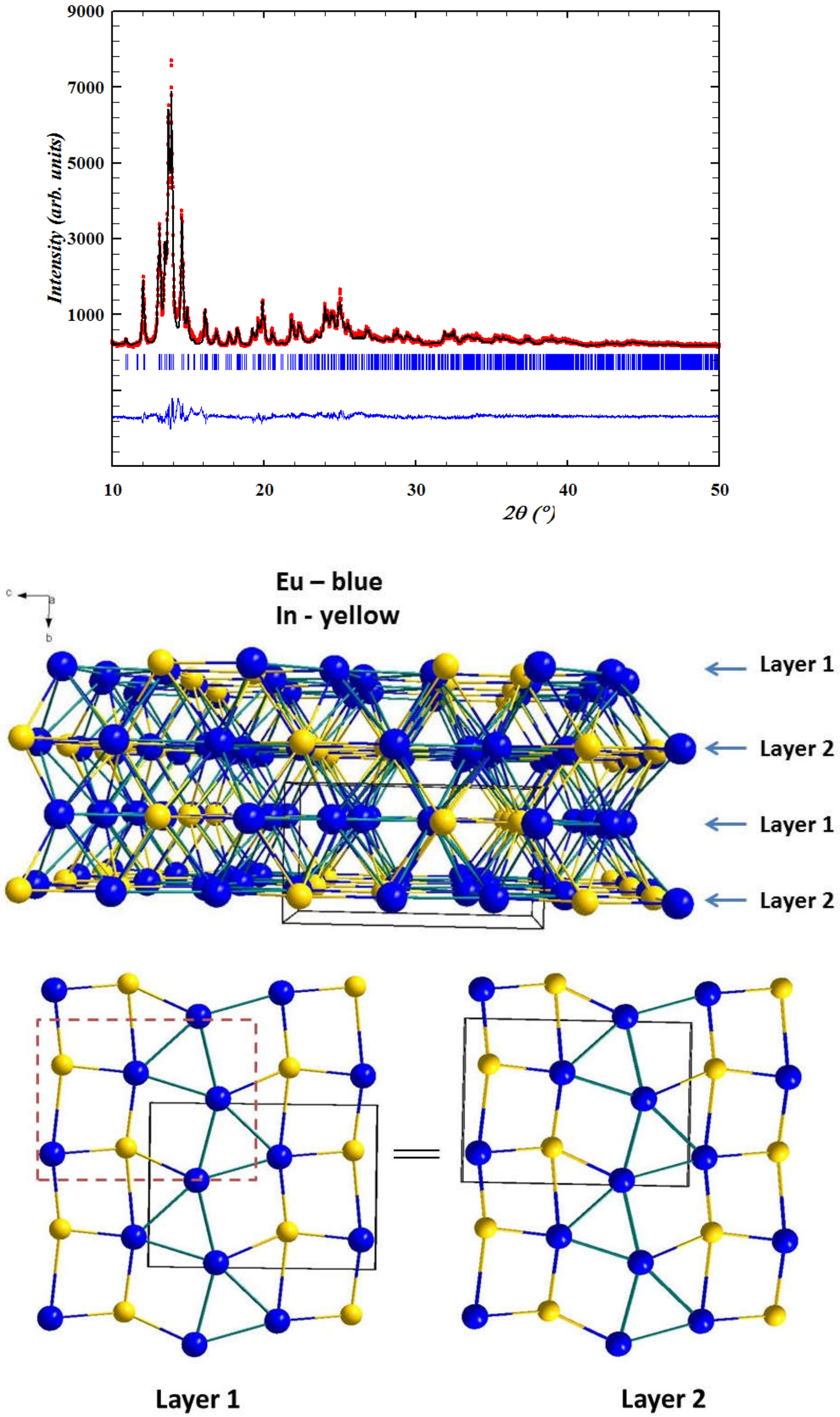

Supplementary Figure 1 Powder XRD and crystal structure of Eu_2In. Top, powder XRD from Mo K_α source and Rietveld refinement at 300 K, observed intensity (symbols), calculated intensity (black line), and difference (bottom blue line). Middle, schematic 3D representation illustrating the crystal structure of Eu_2In. Bottom, cut of the different layers illustrating the relationship between them.

Supplementary Note 2: Powder x-ray diffraction as a function of the temperature

Powder x-ray diffraction data as a function of temperature were acquired using Mo K_{α} Rigaku TTRAX diffractometer upon cooling. The Eu_2In material was hand-crushed in a mortar with a pestle and screened (<22 μm) in an Ar-filled glovebox, then mixed with petroleum jelly before mounting on a copper sample holder. As the crushing, the particle size reduction or the solidification of petroleum jelly on cooling, may affect the properties, magnetization measurements were carried out on this XRD sample after the measurements. **Supplementary Figure 2** shows a sharp ferromagnetic transition at 55 K, confirming that the powder used for XRD experiments is representative of the bulk reported in the main article.

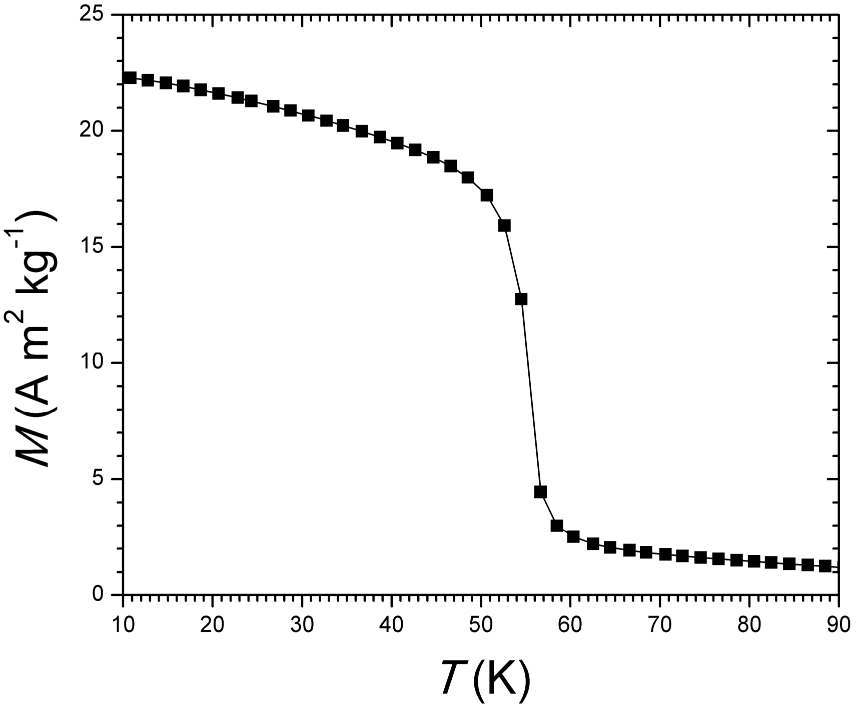

Supplementary Figure 2 Magnetization measurements on a powder XRD sample. Magnetization (*M*) as a function of the temperature (*T*) in 0.1 T for Eu_2In powder (particle size <22 μm) dispersed in petroleum jelly (corrected for mass).

Supplementary Figure 3 illustrates XRD experiments for selected temperatures around the transition temperature. No significant modification of the diffraction pattern was observed between 50 and 60 K. A small but still noticeable shift toward higher Bragg angles can be observed when lowering the temperature, which is most pronounced between 50 and 60 K, *i.e.* at the Curie temperature. Qualitatively, these XRD patterns indicate an isostructural compression of the unit cell when lowering the temperature and across the ferromagnetic transition at 55 K in zero magnetic field.

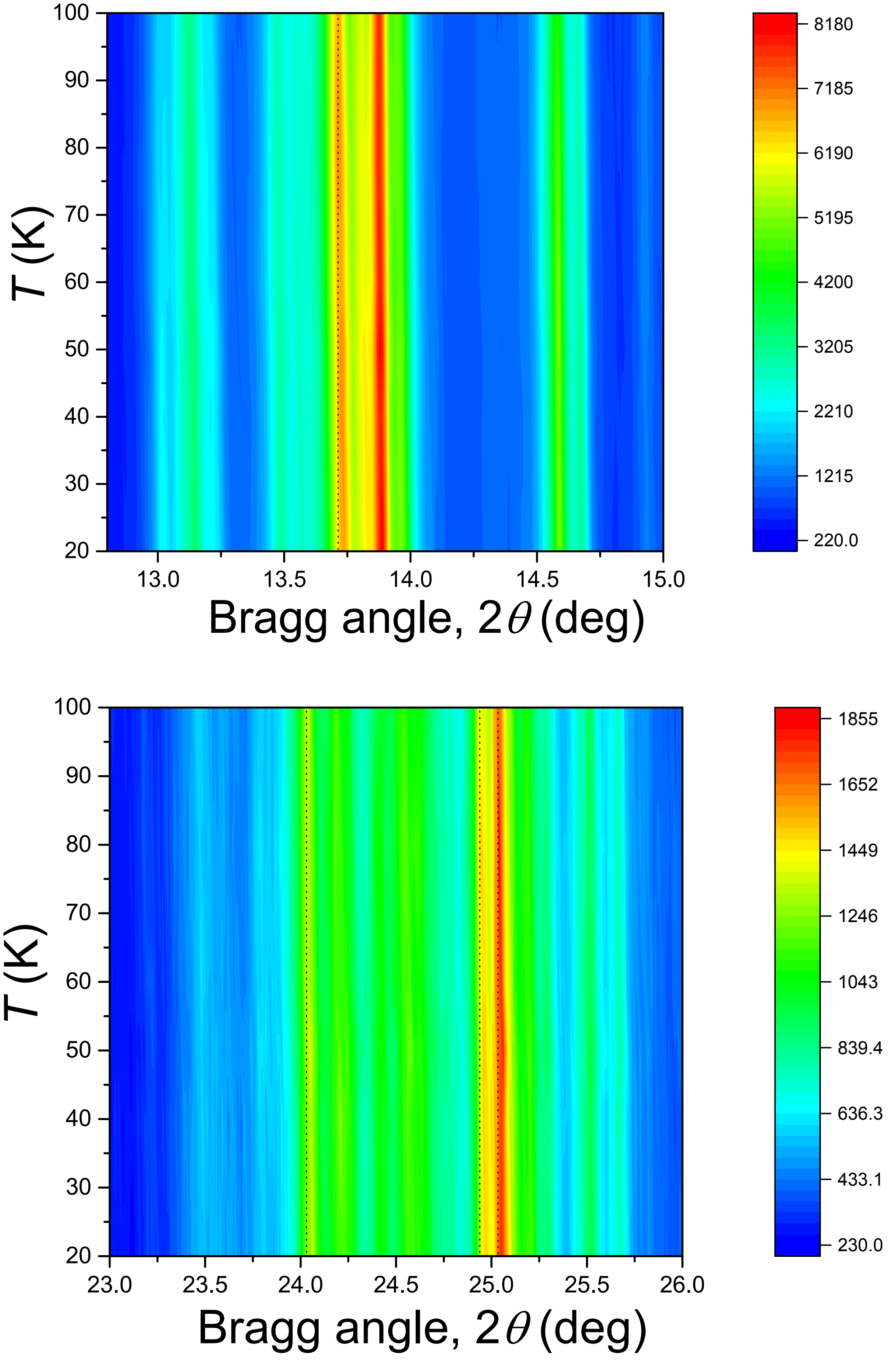

Supplementary Figure 3 Fragments of powder XRD patterns at selected temperature (Mo K_α source). Top, around the most intense diffraction peaks. Bottom, at higher Bragg angles. The vertical dotted lines are guides for the eyes

Supplementary Note 3: AC magnetic susceptibility

AC magnetic susceptibility measurements are shown in **Supplementary Figure 4**. These measurements were carried out upon warming in zero external dc magnetic field after zero field cooling at 6 frequencies (10, 100, 300, 500, 1000 and 1200 Hz) with 5 Oe oscillation amplitude. The long range ordering transition is clearly the most striking feature in both the real and imaginary components. While the real component of AC susceptibility (χ') resembles the DC magnetization data, the imaginary component (χ") shows a sharp peak at the magnetic phase transition. The non-Curie-Weiss behavior in DC magnetization and χ' between T_C = 55 K and about 100 K and a minor yet gradual increase of χ" above 0 may indicate development of short-range correlations above T_C. Neither χ' nor χ" signals present significant frequency dependence.

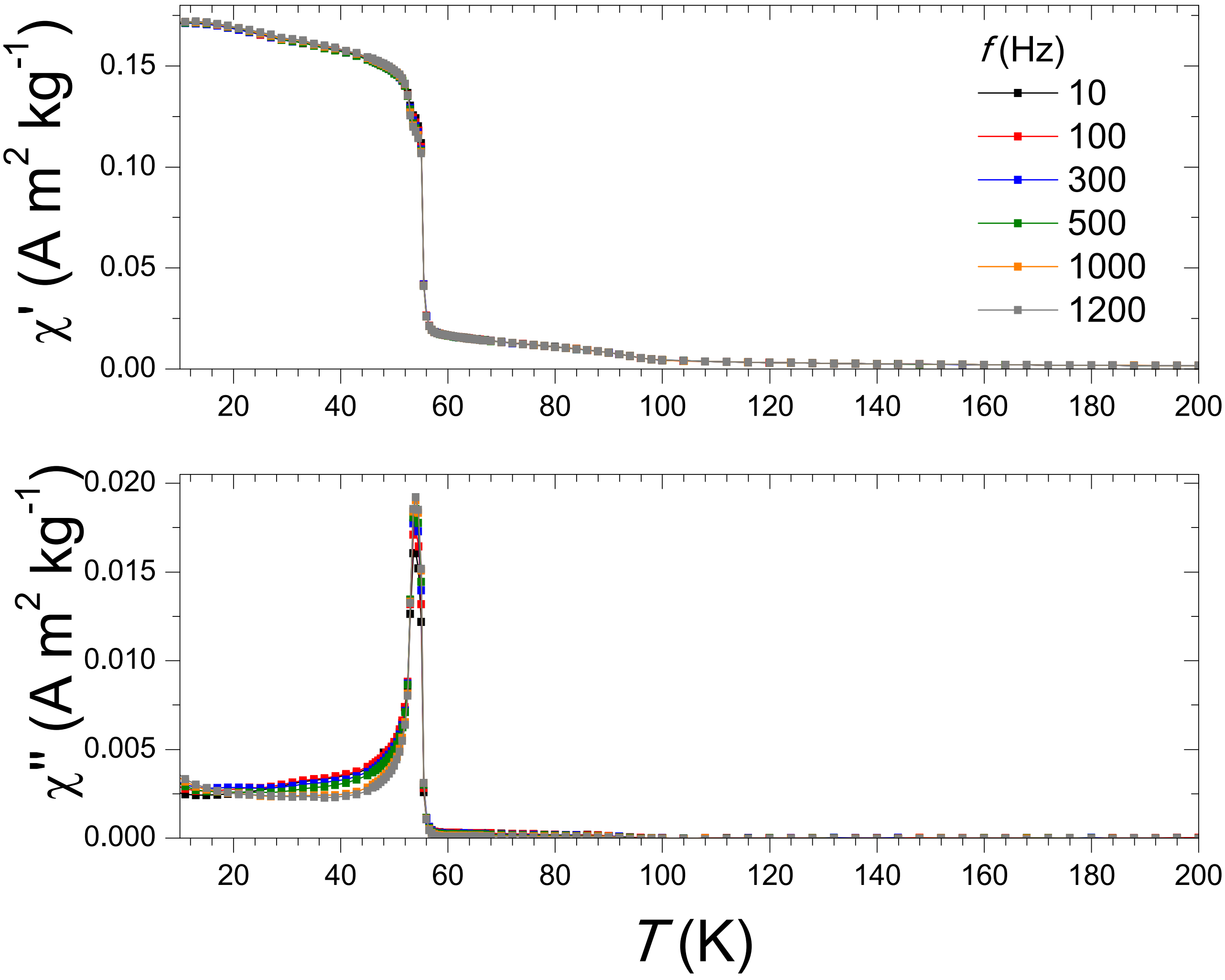

Supplementary Figure 4 AC magnetic susceptibility of Eu_2In. Real (χ') and imaginary (χ'') components of the AC susceptibility in zero external magnetic field for various frequencies (*f*).

Supplementary Note 4: Determination of the thermal hysteresis from magnetization measurements

In Eu_2In, the extreme sharpness of the ferromagnetic transition in combination with an exceptionally small thermal hysteresis makes it experimentally difficult to accurately determine the thermal hysteresis from magnetization measurements. For heat capacity measurements, the thermometer of the measurement platform is in direct contact with the sample, so that it provides an almost instantaneous reading of the sample temperature allowing an accurate determination of the thermal hysteresis at the transition. For resistivity measurements, the sample is in a direct physical contact with the puck and thus with the insert of the cryostat, so that the temperature is well controlled. On the other hand, magnetization measurements suffer from two complications making the use of sweeping techniques challenging: the sample temperature is controlled indirectly through the exchange gas in the insert of the cryostat, and the measurement time is finite (of the order of 10 s for reciprocating extraction over 3 cycles).

In principle, to get magnetization data with an accurate control of the temperature the so-called “settle” or “no-overshoot” modes are recommended, as they allow to stabilize the temperature for each magnetization versus temperature point. Doing so in Eu_2In, in combination with small temperature increments (0.1 K) required to properly describe the transition, results in an inverted hysteresis. In **Supplementary Figure 5** one observes that the heating curves is below the cooling curve around T_C. This unphysical observation is due to the extremely narrow width of the thermal hysteresis in Eu_2In and the transition itself, which initiates and completes within about 1 K temperature window. During the stabilization process to a new lower (higher) temperature, the insert of the cryostat endures a small overshooting toward a lower (higher) temperature than the target, and then stabilizes through decaying thermal oscillations around the target temperature. In the present case, the thermal hysteresis is as small or smaller than the overshooting and oscillation process, so that the thermal hysteresis cannot be determined using these so-called “settle” or “no-overshoot” modes.

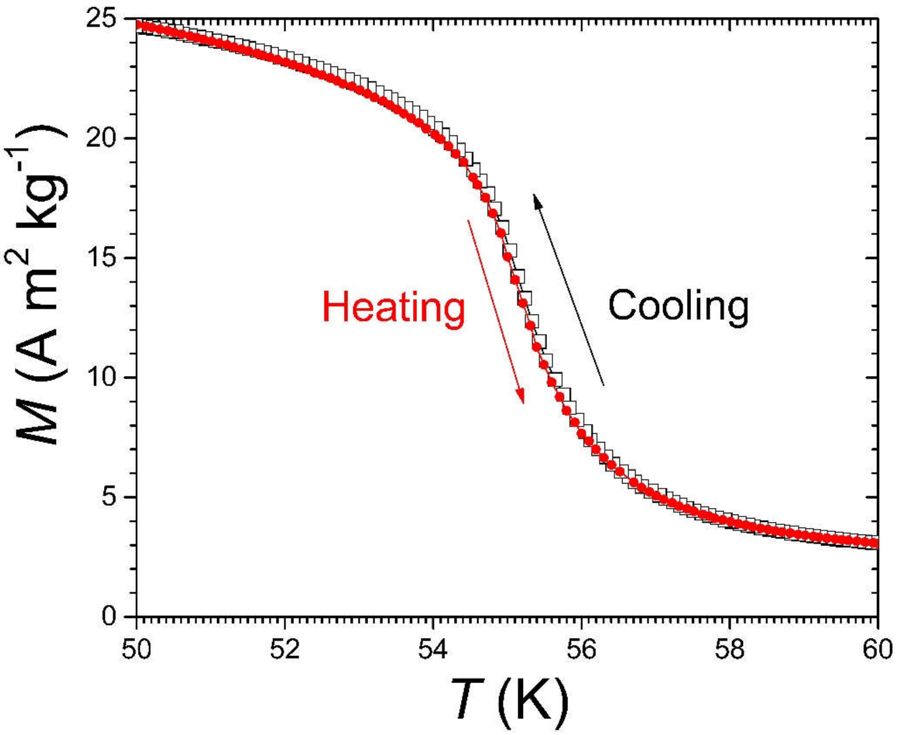

Supplementary Figure 5 Magnetization of Eu_2In. Magnetization (*M*) measurements as a function of the temperature (*T*) in *B* = 0.1 T upon cooling and upon heating using a stabilization of the temperature at each point (so-called "settle" or "no-overshoot" modes).

To avoid thermal stabilization issues, one has to use temperature sweep methods, i.e. measurements during a continuous change in temperature. Such an approach however introduces a temperature lag between the insert of the cryostat and the sample. A practical method, common in differential scanning calorimetry measurements, may still allow a determination of the thermal hysteresis free from thermal lag. It consists of measuring the physical property of interest at different sweeping rates and extrapolating the results toward static conditions, i.e. to a zero sweep rate. For that purpose, the magnetization of Eu_2In was measured using different sweeping rate between 2 K min^{-1} to 0.1 K min^{-1} upon heating and upon cooling in an applied magnetic field of 0.1 T. The Curie temperature is defined as the maximum in the absolute value of the derivative of magnetization with respect to temperature. The transition temperatures upon heating and cooling are shown in **Supplementary Figure 6**. This approach allows us to obtain a reliable estimate of the thermal hysteresis from magnetization data of about 0.07 K, which is now in line with heat capacity and resistivity measurements.

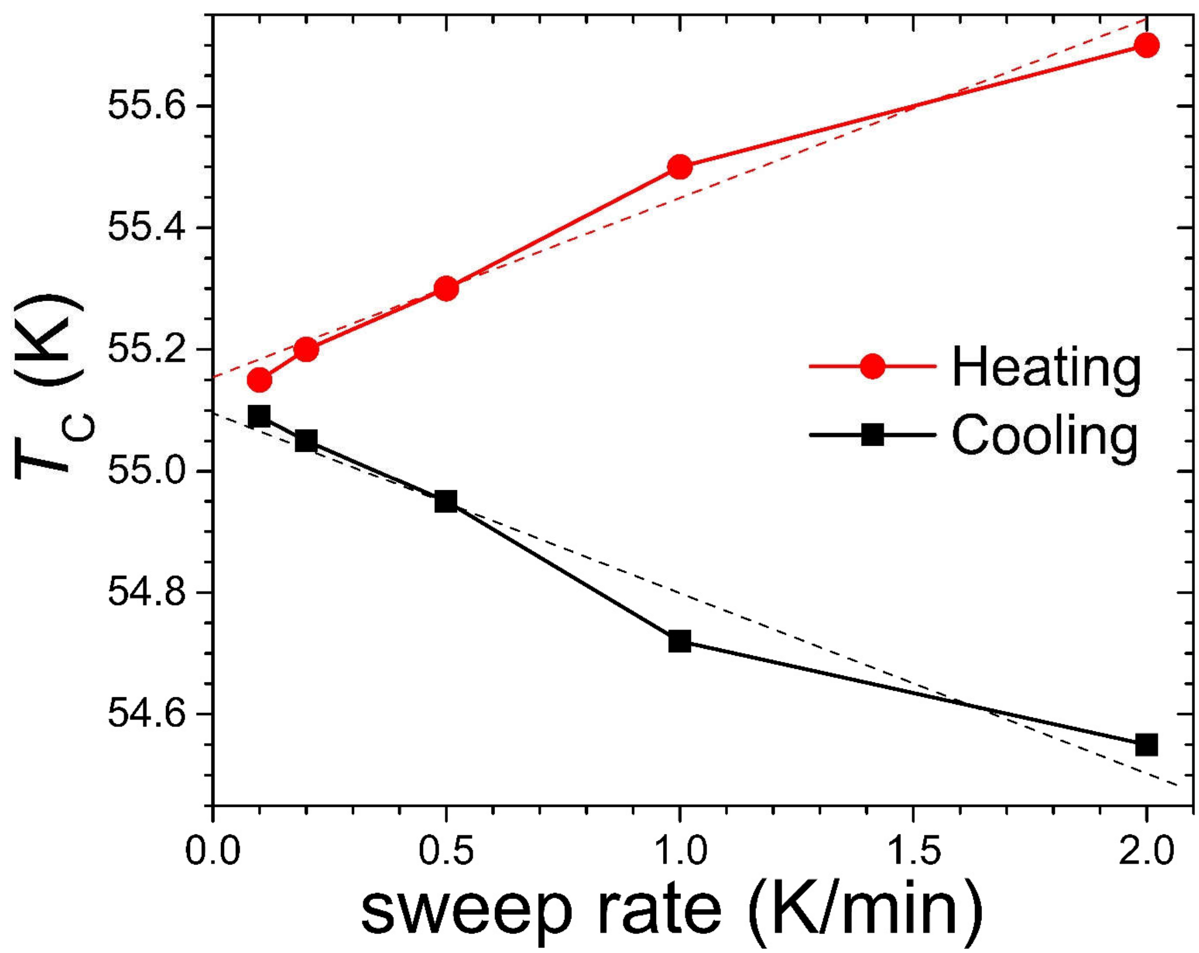

Supplementary Figure 6 Determination of the Curie temperature (T_C) from magnetic data. Ferromagnetic transition temperatures of Eu_2In in B = 0.1 T measured upon cooling and upon heating using magnetization data recorded at different sweeping rate.

Supplementary Note 5: Magnetization at high pressure

A piece of lead wire was used as internal manometer for the magnetization measurements. The superconducting temperature of Pb was used to translate into pressure, P = -0.379*(T-T_0), where T is the superconducting transition temperature with applied pressure and T_0 is the same at zero pressure (7.18 K). The superconducting temperature was determined by AC magnetic susceptibility measurements, since it corresponds to a well-defined peak in the imaginary part of the susceptibility. The measurements were carried out by progressively increasing the pressure. **Supplementary Figure 7** illustrates the determination of the pressure.

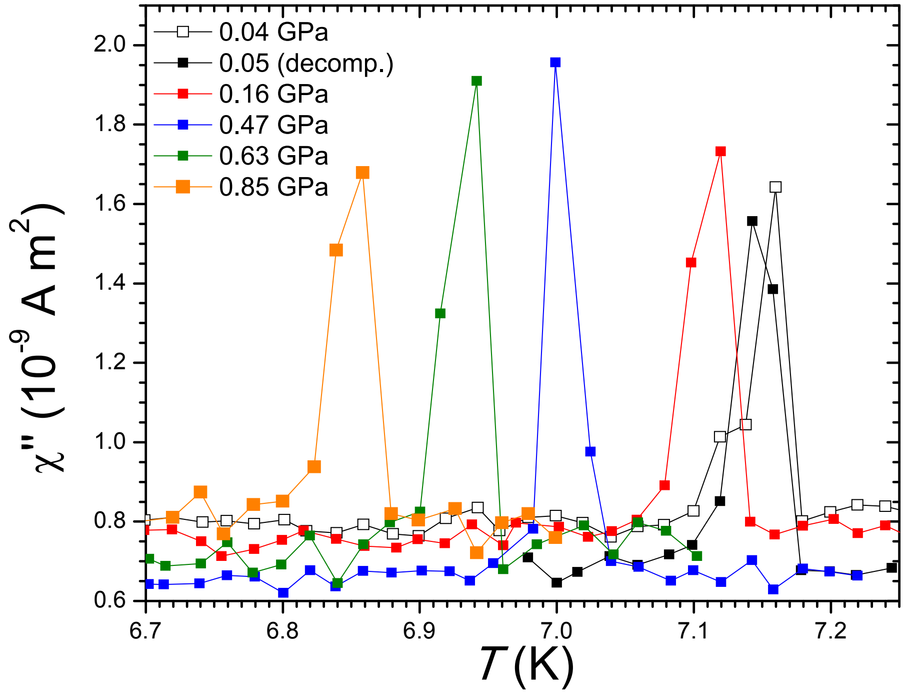

Supplementary Figure 7 Pressure determination for magnetic measurements. Temperature (T) dependence of the imaginary component (χ'') of the AC magnetic susceptibility for the lead piece used as a manometer.

Besides the magnetization versus temperature measurement under pressure presented in the manuscript, additional measurements were performed and are presented in **Supplementary Figure 8**. First, to ensure that the small dT_C/dP does not originate from an artefact, the low pressure (hand tightening of the cell) properties were measured before the compression and after decompression. The very good reproducibility of the magnetic properties observed in Supplementary Figure 8a provides support to the reliability of the high pressure magnetic experiments.

Then, magnetization measurements at T = 10 K do not show any evolution of the saturation magnetization, Supplementary Figure 8b, indicating that the divalent character of the Eu atoms is robust.

Finally, magnetization measurements were performed near the ferromagnetic transition under 0.85 GPa. Qualitatively, the behavior is similar to that observed in zero pressure. However, at high pressure (0.85 GPa) the dT_C/dB =4.7 K T^{-1} is larger than at zero pressure, indicating a certain weakening of the first-order transition when applying the pressure. This effect is related to the usual broadening of any ferromagnetic FOMT when brought out to higher temperature by an external parameter (either magnetic field or pressure).

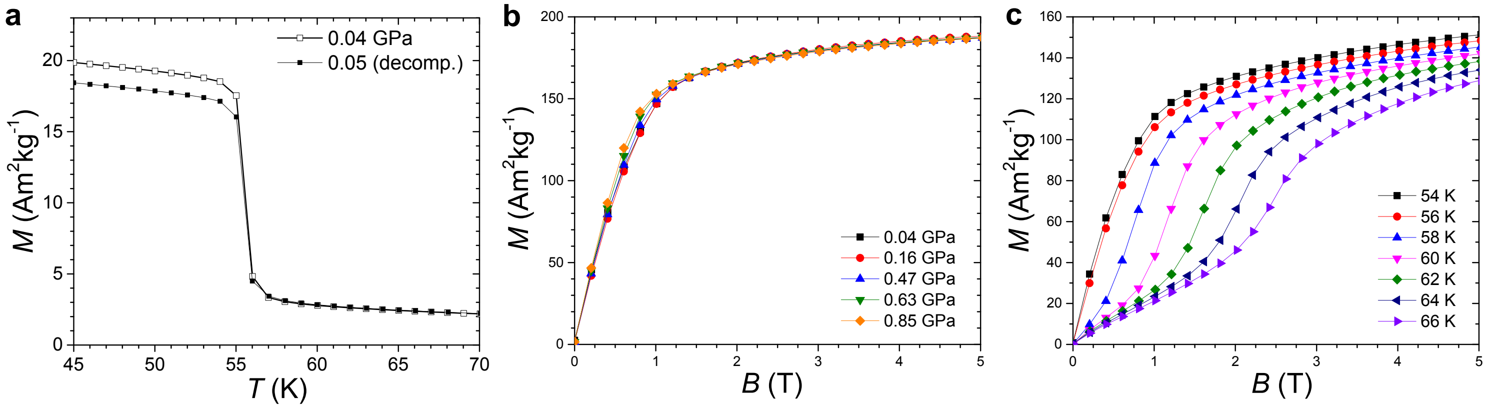

Supplementary Figure 8 Complementary magnetic measurements at high pressure. (**a**) Magnetization (*M*) *versus* temperature in 0.1 T measured with the high pressure cell before and after compression. (**b**) Magnetization curves at $T = 10$ K as a function of the applied pressure. (**c**) Set of magnetization curves in 0.85 GPa around the ferromagnetic transition.

Supplementary Note 6: Electrical resistivity near the first-order magnetic transition

Due to the difficult extraction of large Eu_2In pieces out of the tantalum ampule, a special sample was prepared for resistivity measurements (ρ). Eu_2In chunks were crushed into a fine powder, then cold-pressed in a die into a pellet with 10 mm diameter. The pellet is thereafter sealed in a quartz ampule backfilled with low pressure of He, and sintered at 500 °C for 24 h. The resulting sintered bulk piece is grey metallic to the eye, but examination under an optical microscope reveals a significant porosity. Part of the pellet is cut for resistivity measurements. The electrical resistivity is measured using a standard 4-point technique and the AC transport option of a 14 T physical properties measurement system (PPMS, Quantum Design). Copper wires were attached to the sample using silver containing epoxy. All preparation steps of the resistivity measurements (shaping of the pellet, mounting of the sample on the resistivity puck, electrical contacts…) were carried out in a purified argon glovebox.

The resistivity measurements were repeated at each temperature point using both AC and DC excitations, and using different current amplitudes in the range 20 to 100 mA. The resistivity values are found to present a very limited dependence on the measurement parameters. The results obtained using DC excitation with 100 mA are shown in **Supplementary Figure 9**. Two sets of measurements were carried out, a first one using 2 K temperature increments, panel a, and a second centered on the transition with 0.1 K temperature increments, panel b. The first-order transition clearly manifests itself as a step in resistivity separating two rather linear regimes. Above the ferromagnetic transition, one observes a linear increase of the electrical resistivity with the increasing temperature which is typical of a metallic material. The development of a magnetic order below T_C = 55 K leads to a much steeper slope of the $\rho(T)$ curve due to a combination of electron-phonon and much-reduced electron-magnon interactions. Not only there is an excellent agreement of the transition temperature (55.2 K) with magnetization and heat capacity measurements, but the very small thermal hysteresis (0.1 K) is also confirmed by resistivity. Due to the sharpness of the ferromagnetic transition and its sensitivity to the magnetic field, a sizable magnetoresistance is fully expected in Eu_2In.

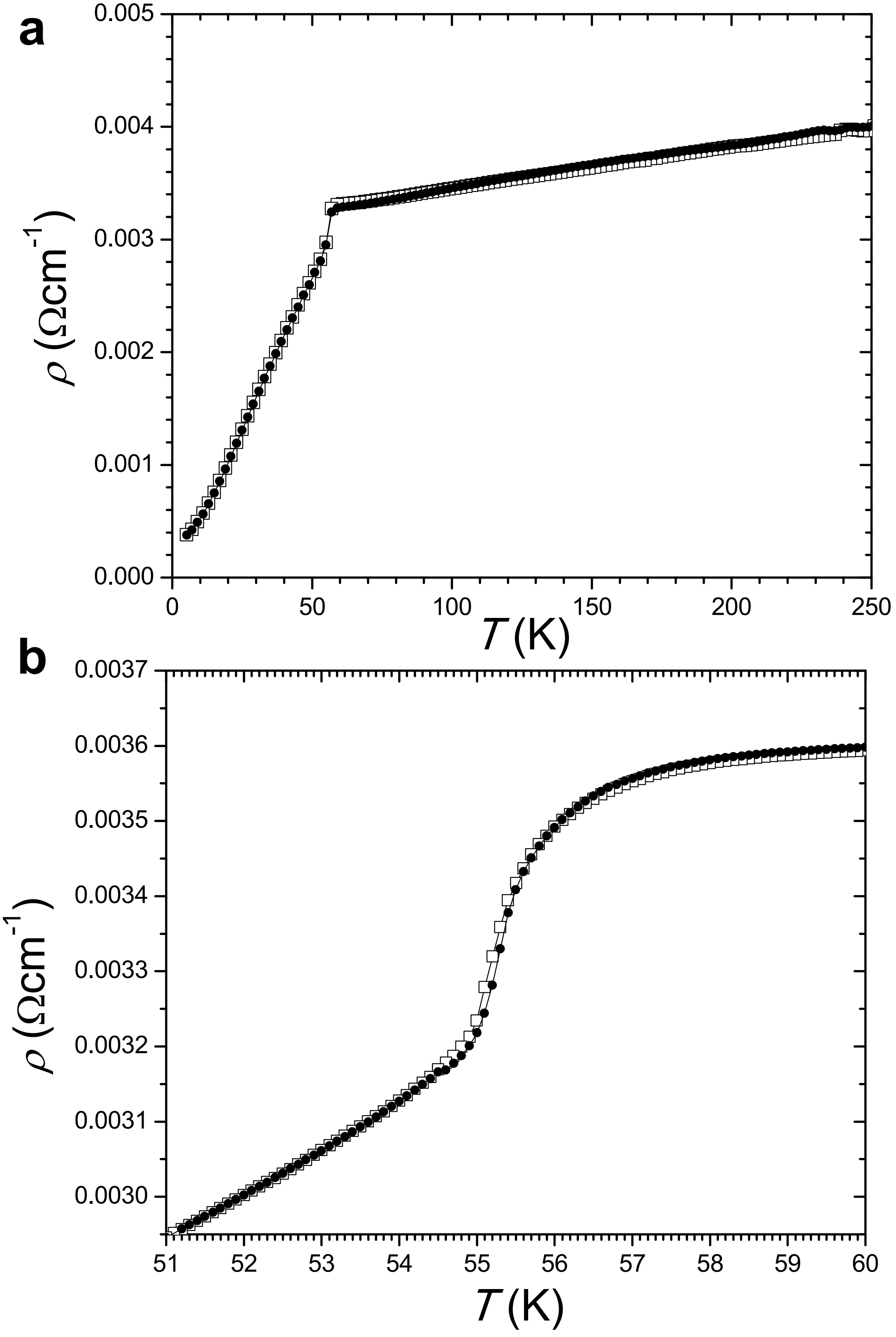

Supplementary Figure 9 Electrical resistivity of Eu_2In. Electrical resistivity (ρ) measured in $B = 0$ upon cooling (squares) and upon heating (circles). Panel (**a**) measurements with 2 K temperature increments, (**b**) second set of measurements with 0.1 K temperature increments.

Supplementary Note 7: Single pulse method (SPM) for heat capacity measurements

The heat-pulse semi-adiabatic method is at present one of the most common ways to measure the heat capacity. The commercial system, as implemented in the Physical Properties Measurement System (PPMS, Quantum Design) is one of the most frequent setups for such measurements. This equipment is convenient to use and is reliable as the relative accuracy is typically 1 % in the temperature range 5-300 K. This technique is however also known to suffer from major issues around extremely sharp heat capacity peak, or in case of thermal hysteresis. Here to address this issue we used the method proposed by V. Hardy which consists in crossing the FOMT in a single temperature pulse[1]. This leads to a raw temperature-time signal as shown in **Supplementary Figure 10**. The FOMT manifests itself by plateaus on the $T(t)$ curve, one at about 55.3 K on the temperature rising branch (heater power ON), and another about 55.2 K on the cooling branch (heater OFF). From this relaxation curve a heat capacity upon heating and upon cooling is built for the temperature range covered by the single pulse. The final heat capacity curve as presented in the main text is obtained by replacing the usual analysis from the Quantum Design software by the single pulse data upon heating in the temperature range 54 – 56 K. The inset of Figure 3a in the main text illustrates the usefulness of the Single Pulse Method to both obtain the sufficient temperature resolution and differentiate between the heating and cooling branches required to fully describe the heat capacity peak.

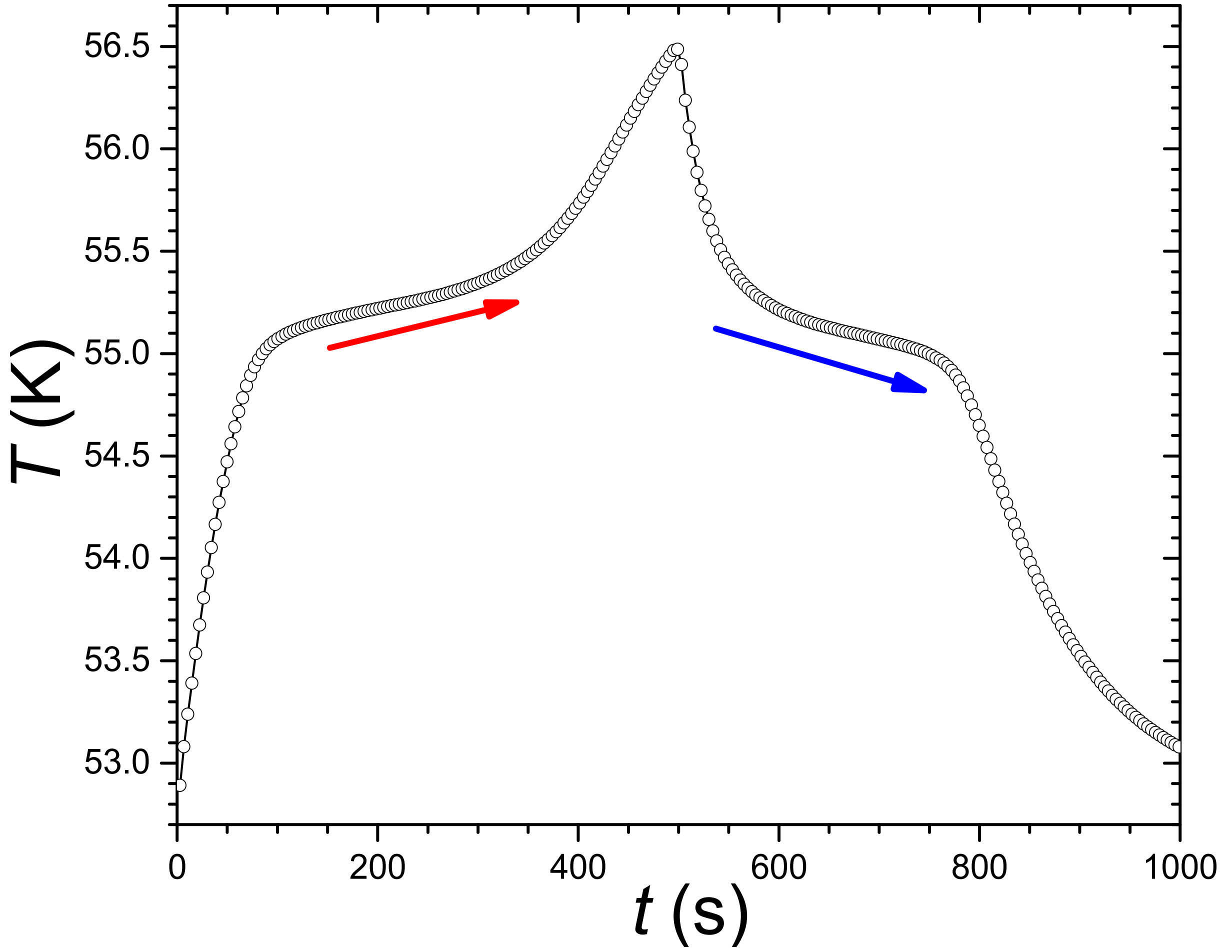

Supplementary Figure 10 Heat capacity relaxation. Temperature (T) versus time (t) relaxation curve of a single pule across the FOMT in B = 0, heating and cooling branch. The arrows mark the transition upon heating (red) and cooling (blue).

Supplementary Note 8: High magnetic field calorimetric and magnetocaloric measurements

In order to derive the magnetocaloric quantities, heat capacity and magnetization measurements were carried out in finite magnetic fields. The heat capacity curves in 1 and 2 T magnetic fields are shown in **Supplementary Figure 11**. As expected for a ferromagnet, with the increase of the magnetic field the heat capacity peak shifts to higher temperature and broadens. It results in a heat capacity peak which can no longer be fully covered by a single pulse, but this technique nevertheless allows an accurate determination of the maximum heat capacity of the peak even in 1 and 2 T.

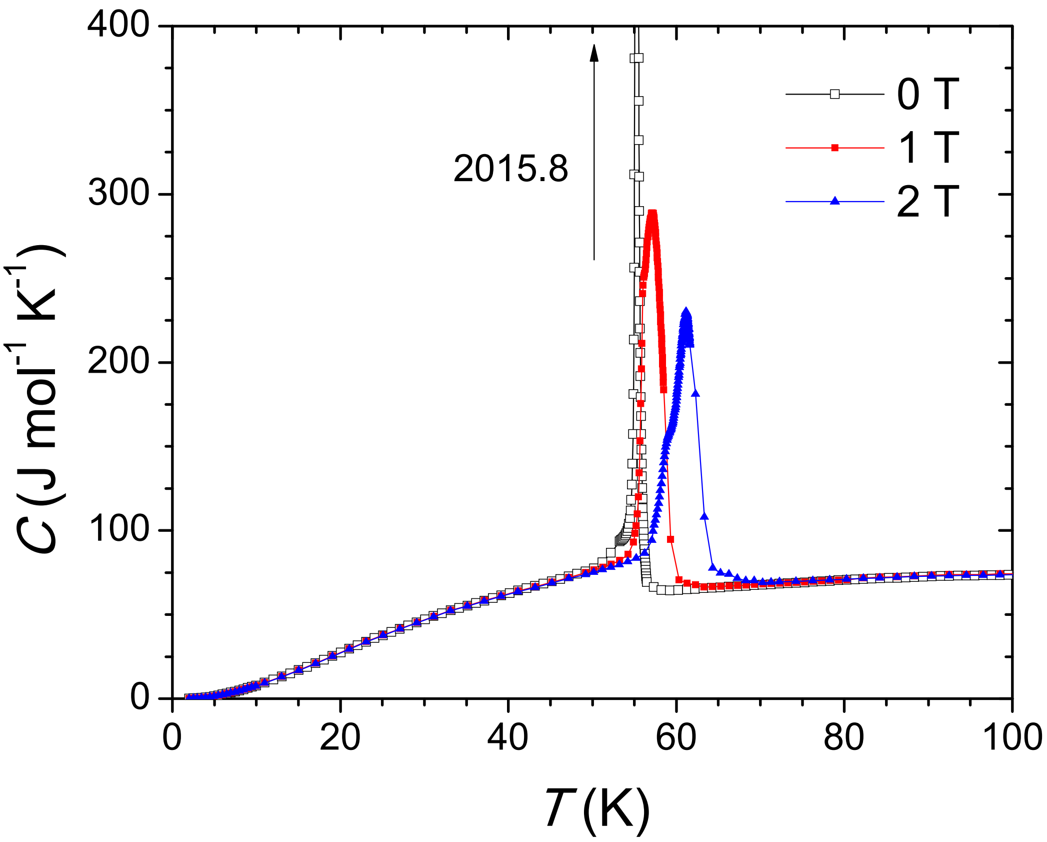

Supplementary Figure 11 Heat capacity data in finite magnetic field. Heat capacity in 0, 1 and 2 T measured upon heating.

For magnetic fields larger than 2 T, the isothermal entropy changes (magnetocaloric effect, ΔS) were determined from isofield magnetization measurements, *i.e.* magnetization versus temperature curves recorded at different magnetic fields. The entropy changes are then calculated using Maxwell equation[2], which are shown in **Supplementary Figure 12** in fields up to 7 T. At high magnetic fields, the maximum ΔS tends to slowly

saturate above 2 T (ΔS_{max} is -37 J kg^{-1} K^{-1} in 7 T), and the ΔS(T) curves develop a tower-like shape whose width in temperature increases with the field, as usual for a ferromagnetic FOMT.

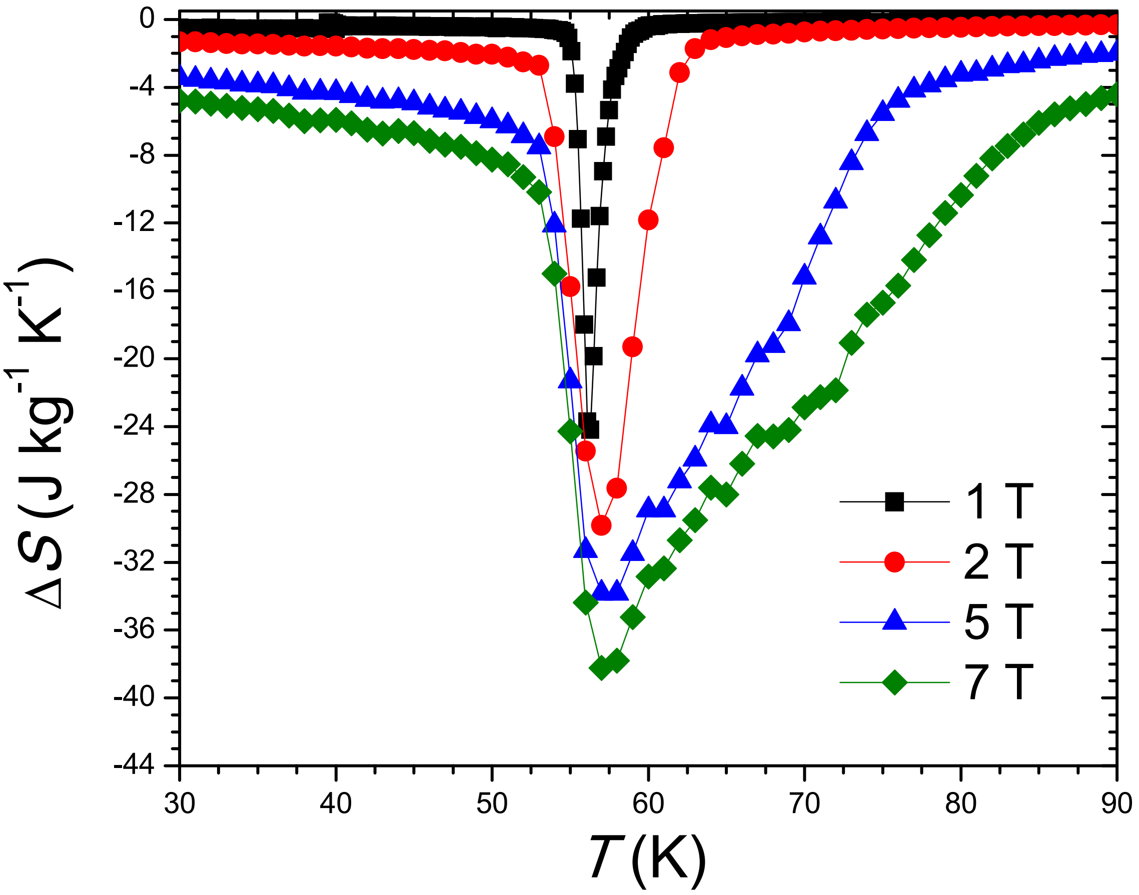

Supplementary Figure 12 Magnetocaloric entropy change in different magnetic fields. Isothermal entropy change (ΔS) determined from isofield magnetization measurements upon heating with temperature increment of 0.2 K for B = 1 T and 1 K for $B \geq 2$ T.

Supplementary Note 9: Temperature-dependent x-ray absorption and field-dependent x-ray magnetic circular dichroism measurements

X-ray absorption measurements at the Eu L_3 edge were carried out at several temperatures from 300 to 5 K, and are shown in **Supplementary Figure 13**. All spectra perfectly overlap, and remain typical of divalent europium in this temperature range.

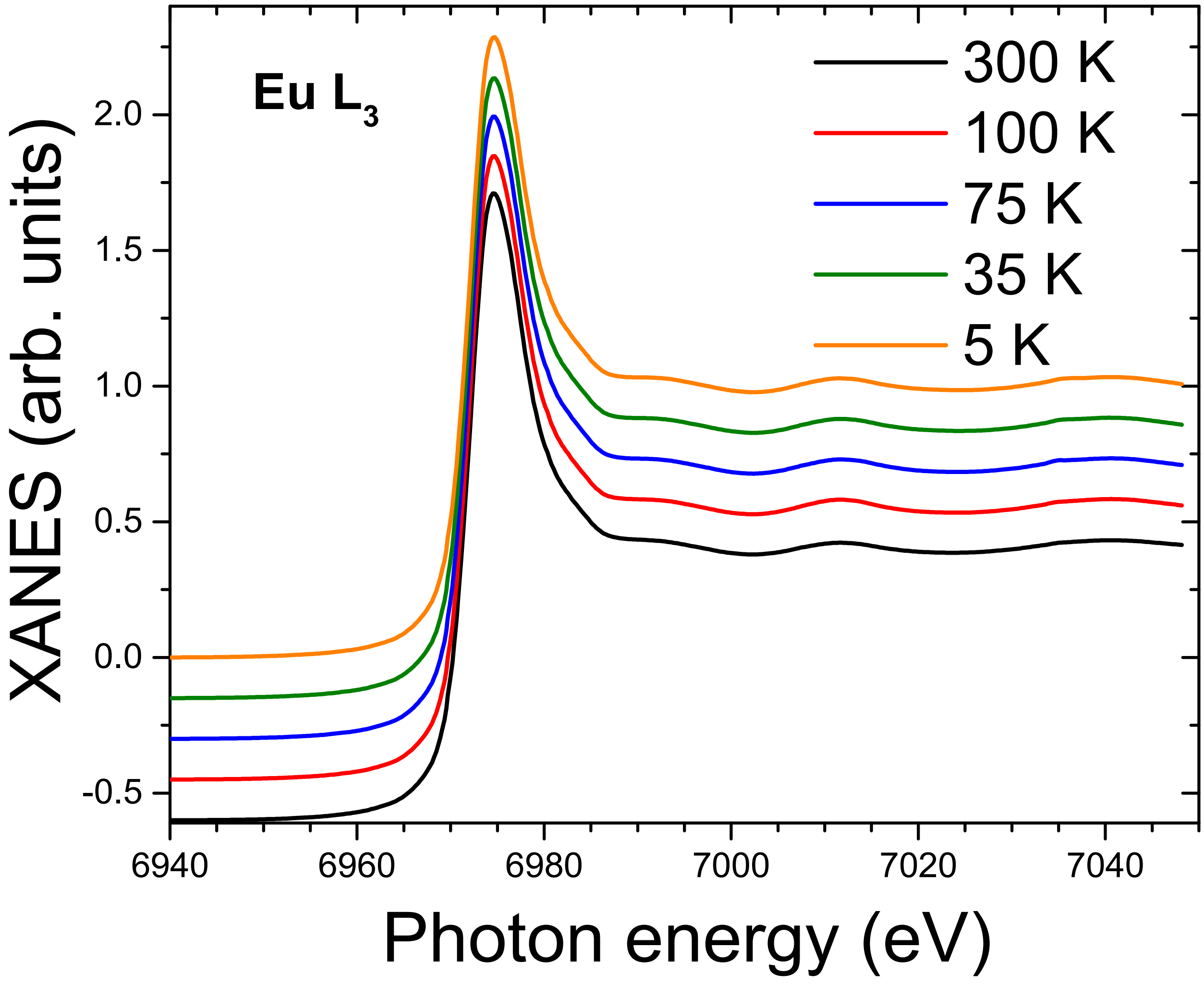

Supplementary Figure 13 Europium valence as a function of the temperature. X-ray absorption near edge (XANES) spectra at the Eu L_3 edge as a function of the temperature (an offset in intensity is added for clarity).

To confirm that the x-ray absorption and magnetic circular dichroism data are truly representative of the bulk properties of Eu_2In and at magnetic saturation, the field dependence of the XMCD was measured at the Eu L_2 edge at T = 5 T up to B = 17 T. The results are shown in **Supplementary Figure 14** and are typical of a ferromagnetic material below its Curie temperature. The shape of the XMCD *vs* B signal is fully in line with the bulk magnetization measurements presented in the main text.

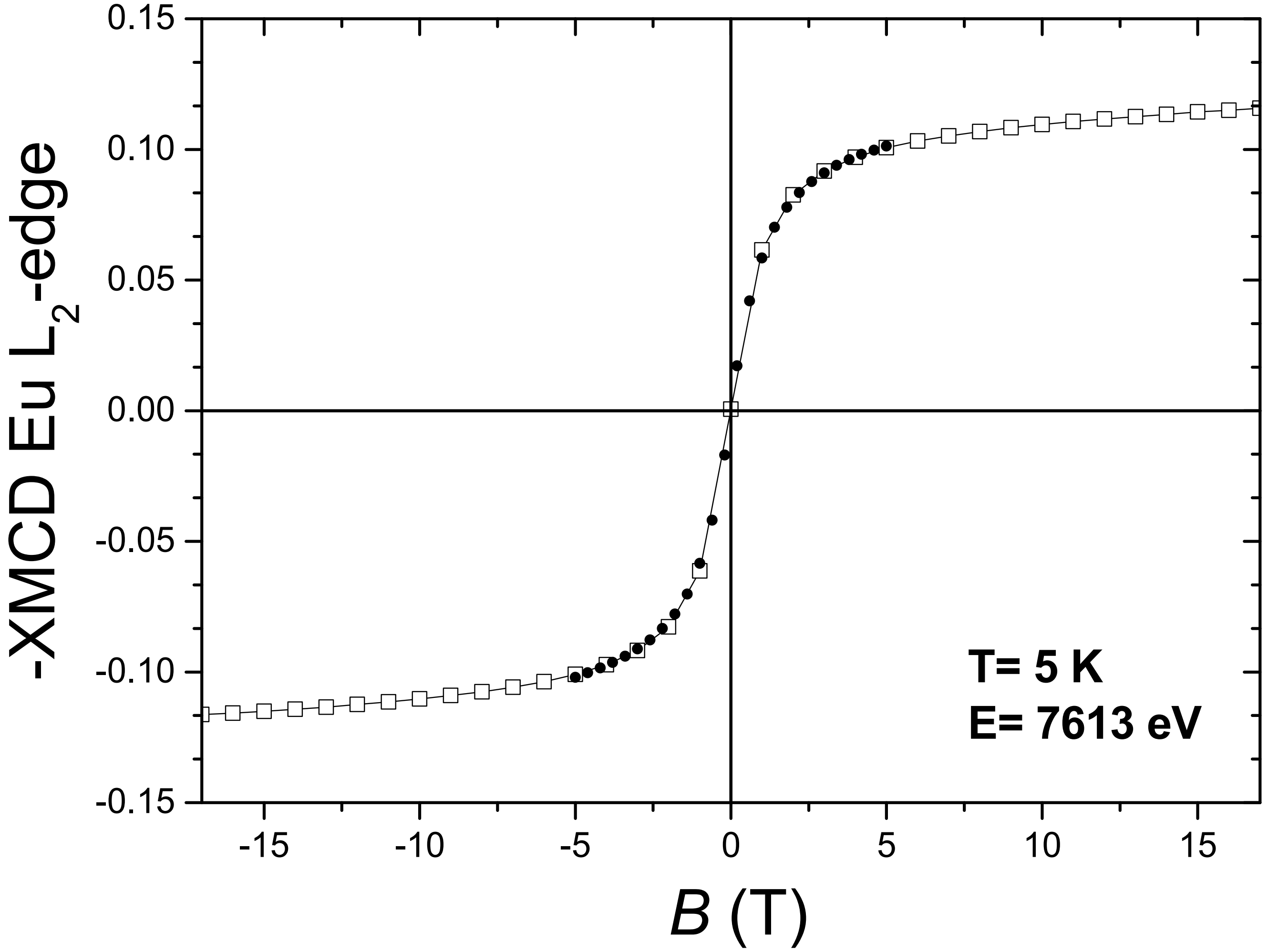

Supplementary Figure 14 Field dependence of the XMCD. X-ray magnetic circular dichroism (XMCD) at the Eu L_2 edge at T = 5 K and up to B = 17 T, the open squares and filled circles indicate two sets of data with different field increments and range.

Supplementary Note 10: Eu_2In antiferromagnetic electronic structure calculations

Since there are two inequivalent Eu sites with identical multiplicities in Eu_2In, the paramagnetic state can be approximated by assuming antiparallel moments of Eu-I and Eu-II sublattices. This arrangement leads to a small (~0.05 μ_B (unit cell)$^{-1}$) net magnetic moment. The corresponding densities of states and magnetic moments are shown in **Supplementary Figure 15** and **Supplementary Table 2**, respectively. The calculations show local 4f (~7 μ_B atom^{-1}) and 5d (~0.1 μ_B atom^{-1}) moments for each of the two independent Eu atoms, however, the net total magnetization is zero. The absence of induced In moments in these approximated paramagnetic calculations is in stark contrast with the results observed for FM Eu_2In presented in the main text.

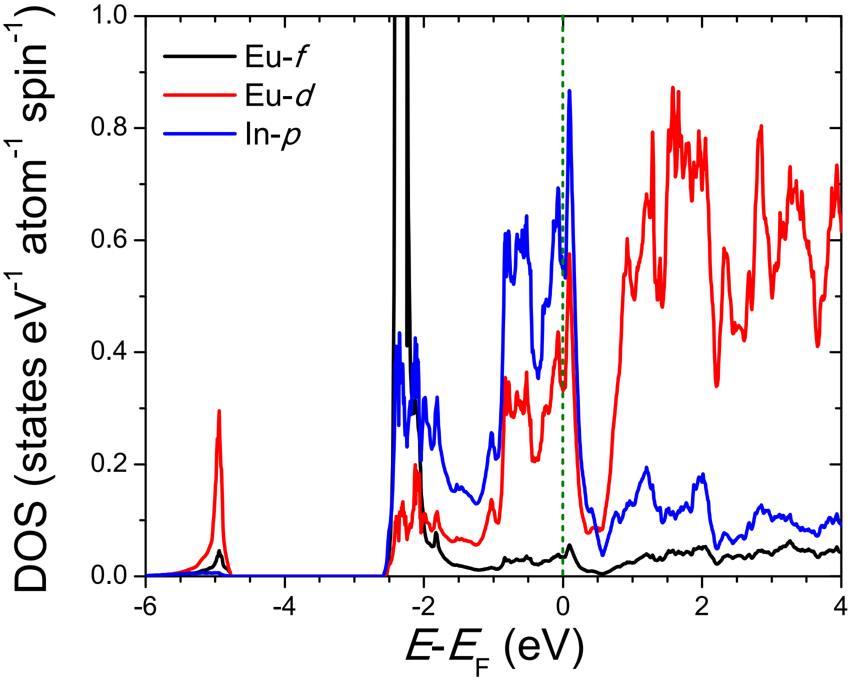

Supplementary Figure 15 Predicted electronic structure of antiferromagnetic Eu_2In. Density of states (DOS) of Eu-I within the approximation of antiparallel Eu-I and Eu-II moments.

Eu_2In (AFM)	s	p	d	f
Eu-I	0.03	0.01	0.09	6.96
Eu-II	-0.01	-0.01	-0.07	-6.94
In	0.00	-0.01	0.00	

Supplementary Table 2 Predicted magnetic moments in antiferromagnetic Eu_2In. Magnetic moment (in μ_B atom^{-1}) for antiferromagnetic Eu_2In (approximation for PM).

Supplementary Note 11: Electronic structure calculations and experimental results for Eu_2Sn

For comparison, a closely related Eu_2Sn was investigated. Eu_2Sn was previously known to crystalize in the orthorhombic Co_2Si-type structure[3]. There is thus a close relationship between Eu_2In and Eu_2Sn as their differences mainly originate from the ratio of their cell parameters (*c/a* or *b/a*). Eu_2Sn was synthesized using the same method as Eu_2In. From powder x-ray diffraction, the structural parameters were found in line with the original report[3]. The magnetic properties of Eu_2Sn are illustrated in **Supplementary Figure 16**. An anomaly typical of an antiferromagnetic transition is observed at T_N = 31 K. A linear fitting of the inverse susceptibility in the temperature range 50 - 330 K indicates an effective paramagnetic moment of 7.94 μ_B Eu^{-1}, pointing toward a divalent Eu state in Eu_2Sn (as in Eu_2In). The application of a magnetic field at low temperature shows a linear behavior typical of a stable antiferromagnetic order.

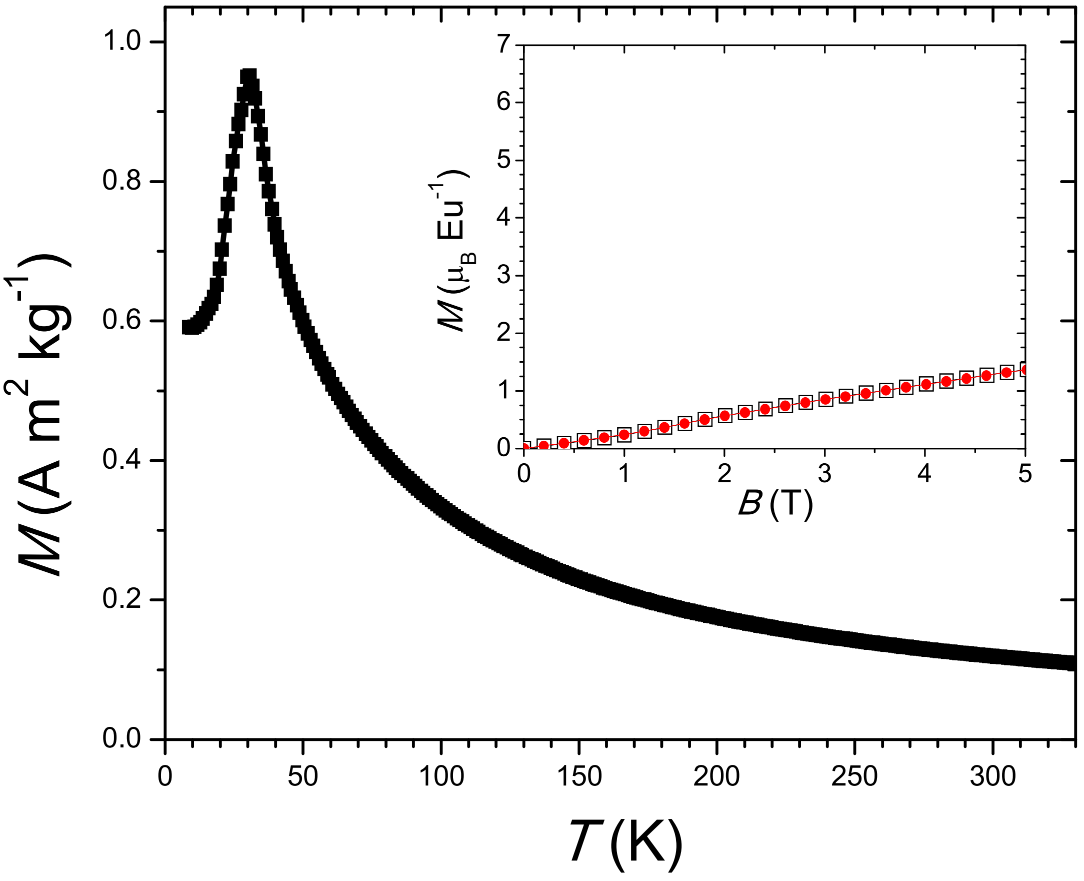

Supplementary Figure 16 Magnetic properties of Eu_2Sn. Magnetization (*M*) as a function of the temperature (*T*) measured in an applied magnetic field *B* = 0.1 T. In inset: magnetization as a function of the magnetic field at *T* = 5 K (magnetization = open symbols, demagnetization = closed symbols).

To further understand the influence of the *p* electrons of In in Eu_2In, we performed electronic structure calculations in Eu_2Sn for which there is one additional *p* electron per formula unit. This additional electron pushes the DOS towards the occupied side, placing the Fermi level in a deep pseudo gap. In contrast, this pseudo gap was at +0.5 eV above the Fermi level in Eu_2In. The ferromagnetic calculations for Eu_2Sn shown in **Supplementary Figure 17** lead to an estimate of the magnetic moment of: 7 μ_B of Eu-4f, a small spin polarized magnetic moments of Eu-5d (0.07 μ_B) and Sn-5p (-0.09 μ_B). This small but non negligible negative Sn-5p moment in the ferromagnetic calculations indicates an unstable ferromagnetic state in Eu_2Sn. The magnetic moments for Eu_2Sn are presented in **Supplementary Table 3** for ferromagnetic and antiferromagnetic (antiparallel EuI-EuII) configurations. The amplitudes of the magnetic moments in Eu_2Sn do not appear significantly influenced by the magnetic configuration. In contrast to Eu_2In, no significant moments develop on either Eu-5d or Sn-4p states. Total energy calculations favor antiferromagnetic state as the ground state, in full agreement with the experimental results shown in supplementary figure 15.

Eu_2Sn (AFM)	s	p	d	f
Eu-I	0.02	0.01	0.07	6.97
Eu-II	-0.02	-0.01	-0.07	-6.94
Sn	0.00	-0.03	0.00	
Eu_2Sn (FM)	s	p	d	f
Eu-I	0.02	0.02	0.07	6.97
Eu-II	0.02	0.00	0.06	6.94
Sn	-0.02	-0.09	0.01	

Supplementary Table 3 Magnetic moments for Eu_2Sn. Magnetic moments (in μ_B $atom^{-1}$) on the different orbitals of antiferromagnetic and ferromagnetic Eu_2Sn.

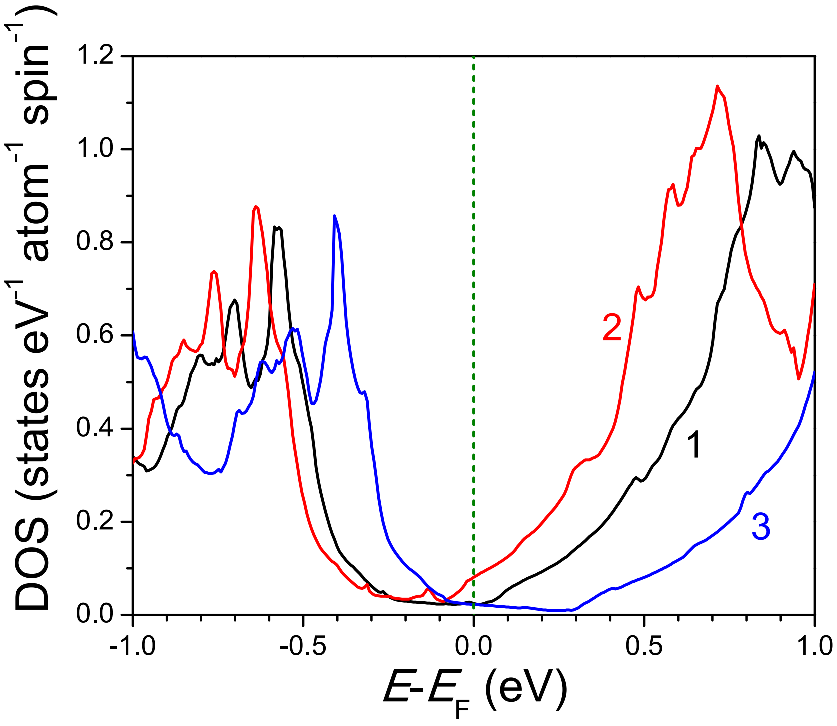

Supplementary Figure 17 Electronic structure of Eu_2Sn. DOS of Eu-I atom (s,p,d,f) around the Fermi level, comparison between the antiparallel EuI-EuII configuration (Up band, 1 black) and forced ferromagnetic configuration for Eu_2Sn (Up, 2 red and Down, 3 blue).

Supplementary References

1. Hardy, V., Bréard, Y. & Martin, C. Derivation of the heat capacity anomaly at a first-order transition by using a semi-adiabatic relaxation technique. *J. Phys.: Condens. Matter* **21,** 075403 (2009).
2. Gschneidner, K. A. Jr, Pecharsky, V. K. & Tsokol A. O. Recent developments in magnetocaloric materials. *Rep. Prog. Phys.* **68,** 1479-1539 (2005).
3. Palenzona, A., Manfrinetti, P. & Fornasini, M. L. The phase diagram of the Eu–Sn system. *J Alloys Compounds* **280**, 211-214 (1998).
